# Discovery and Validation of Novel Methylation Markers in *Helicobacter pylori*-Associated Gastric Cancer

**DOI:** 10.1155/2021/4391133

**Published:** 2021-12-08

**Authors:** Huan Wang, Nian-Shuang Li, Cong He, Chuan Xie, Yin Zhu, Nong-Hua Lu, Yi Hu

**Affiliations:** ^1^Department of Gastroenterology, The First Affiliated Hospital of Nanchang University, Nanchang, 330006 Jiangxi Province, China; ^2^Medical College of Nanchang University, Nanchang, 330006 Jiangxi Province, China

## Abstract

Previous studies have shown that abnormal methylation is an early key event in the pathogenesis of most human cancers, contributing to the development of tumors. However, little attention has been given to the potential of DNA methylation patterns as markers for *Helicobacter pylori*- (*H. pylori*-) associated gastric cancer (GC). In this study, an integrated analysis of DNA methylation and gene expression was conducted to identify some potential key epigenetic markers in *H. pylori*-associated GC. DNA methylation data of 28 H*. pylori*-positive and 168 H*. pylori*-negative GC samples were compared and analyzed. We also analyzed the gene expression data of 18 H*. pylori*-positive and 145 H*. pylori*-negative GC cases. Finally, the results were verified by *in vitro* and *in vivo* experiments. A total of 5609 differentially methylated regions associated with 2454 differentially methylated genes were identified. A total of 228 differentially expressed genes were identified from the gene expression data of *H. pylori*-positive and *H. pylori*-negative GC cases. The screened genes were analyzed for functional enrichment. Subsequently, we obtained 28 genes regulated by methylation through a Venn diagram, and we identified five genes (GSTO2, HUS1, INTS1, TMEM184A, and TMEM190) downregulated by hypermethylation. HUS1, GSTO2, and TMEM190 were expressed at lower levels in GC than in adjacent samples (*P* < 0.05). Moreover, *H. pylori* infection decreased HUS1, GSTO2, and TMEM190 expression *in vitro* and *in vivo*. Our study identified HUS1, GSTO2, and TMEM190 as novel methylation markers for *H. pylori*-associated GC.

## 1. Introduction

*Helicobacter pylori* (*H. pylori*) is a microaerophilic, spiral-shaped gram-negative bacterium that colonizes the stomach and adheres to the gastric mucosa [1]. *H. pylori* has been confirmed to have close relationships with gastritis, peptic ulcer, gastric cancer (GC), and mucosa-related lymphoid tissue lymphoma [2]. *H. pylori* infection is the most definite risk factor for GC [3]. Out of the 952,000 new cases of GC diagnosed worldwide every year, it is estimated that 753,000 (79%) of the recent cases are attributed to *H. pylori* infection [4]. Currently, the specific mechanism of *H. pylori*-induced GC is still unclear. Moreover, specific molecular markers to identify subjects at high risk of *H. pylori*-associated GC need to be explored.

One of the key regulators involved in the environmental response is the methylation of genomic regulatory regions. The methylation of CpG islands in gene promoters is involved in the regulation of gene expression. DNA methylation abnormalities are the main epigenetic mechanism related to gene silencing and play an important role in tumorigenesis [5, 6]. Overall hypomethylation of specific genes is related to genomic instability and the inactivation of tumor suppressor genes [7]. Several studies have indicated that *H. pylori* infection is related to hypermethylation of gene promoters [8]. However, at the whole-genome level, studies on the mechanism of abnormal DNA methylation involved in the carcinogenesis of *H. pylori*-associated GC are still scarce. Moreover, the molecular mechanism of the abnormal methylation of gene regulatory regions involved in the generation and development of GC remains elusive.

This study is aimed at exploring the potential methylation-related gene markers in *H. pylori*-related GC by analyzing the methylation and gene expression data of The Cancer Genome Atlas (TCGA) database, combined with related experiments.

## 2. Materials and Methods

### 2.1. Data Sources

**Figure 1:**
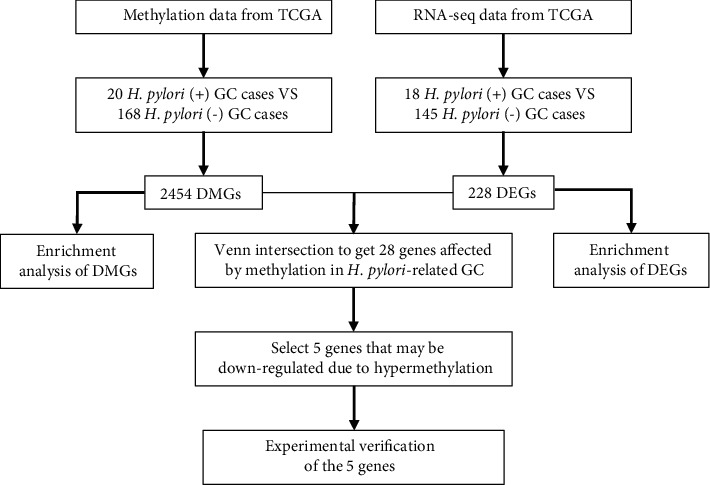
Flowchart of the data acquisition and analysis process. TCGA: The Cancer Genome Atlas (https://cancergenome.nih.gov/); DMGs: differentially methylated genes; DEGs: differentially expressed genes.

DNA methylation data from 397 samples (395 GC and 2 normal samples) were obtained from TCGA (https://cancergenome.nih.gov/). The RNA-Seq data and clinical information of 407 samples (375 tumor samples and 32 normal samples) were obtained from UCSC Xena (https://xenabrowser.net/datapages/). A flowchart of the process of data acquisition and the subsequent analysis is shown in Figure 1.

### 2.2. Screening of Differentially Methylated Genes

After screening, a total of 188 cases with *H. pylori* infection information (20 positive and 168 negative) were included and analyzed. The minfi package [9] was used to preprocess and normalize the DNA methylation data. Benjamini-Hochberg was used to correct the *P* value, and ∣log2FC | >1, *P* < 0.01 were set as the thresholds to screen for differentially methylated sites.

### 2.3. Screening of Differentially Expressed Genes

The samples with clear clinical information of *H. pylori* infection were screened from the tumor samples. A total of 163 GC samples (18 with *H. pylori* and 145 without *H. pylori*) were included in the subsequent analysis. The analysis used the R language edge package [10] to analyze the difference between *H. pylori-*positive and -negative groups. The threshold of differential gene selection was set as *P* < 0.01. The results were visualized by volcano map.

### 2.4. Enrichment Analysis

The genes obtained from differential gene analysis and differential methylation analysis were enriched and analyzed with the R language cluster profiler package [11], and the top 10 genes were used for visualization.

### 2.5. Screening for Decreased Gene Expression due to Hypermethylation

The results of the intersection of the differentially expressed genes (DEGs) obtained from the differential gene analysis and the differentially methylated genes (DMGs) obtained from the differential methylation analysis were taken as the genes affected by the methylation of *H. pylori-*associated GC. In this analysis, the methylation sites of each gene in GC patients infected by *H. pylori* and those not infected by *H. pylori*, as well as the expression of each gene in the two groups, were visualized by box plots, and the Wilcoxon rank-sum test was performed. Ultimately, 5 genes with significantly upregulated methylation levels and significantly downregulated gene expression levels in the *H. pylori*-positive group were screened out.

### 2.6. Cell Culture and *H. pylori* Strains

AGS cells (human GC cells) were cultured in DMEM/F12 (Gibco, CA, USA) containing 10% fetal bovine serum (FBS) and 1% penicillin/streptomycin (Gibco) at 37°C in an atmosphere of 5% CO_2_. *H. pylori* 7.13 (provided by Dr. Richard Peek from the Vanderbilt University Medical Center) was cultured on Campylobacter agar plates containing 10% sheep serum under microaerophilic conditions at 37°C. The concentration of the bacteria was determined by spectrophotometry (OD600 nm) after suspension in DMEM/F12. Subsequently, the cell culture medium was replaced with fresh medium without antibiotics, and the cells were cocultured with *H. pylori* at different times or multiplicities of infection (MOIs).

### 2.7. Western Blotting

The harvested cells were lysed with lysis buffer containing protease inhibitor cocktail (Roche, Amherst, CA, USA). Protein samples were separated by SDS–PAGE and transferred to nitrocellulose membranes. The blocking buffer was prepared with 5% nonfat dry milk in TBST buffer, and the membranes were blocked at room temperature for 1 hour and then incubated with primary antibodies in a shaker at 4°C overnight. Antibodies were anti-GSTO2 (Proteintech, 14562-1-AP), anti-HUS1 (Proteintech, 11223-1-AP), anti-INTS1 (Sigma–Aldrich, HPA021658), anti-TMEM184A (Proteintech, 25989-1-AP), anti-TMEM190 (Invitrogen, PA5-70986), and anti-Actin (TransGen Biotech, HC201-01). The membranes were incubated with the corresponding secondary antibodies (1 : 2000) for 1 hour at room temperature the next day, and finally, the proteins were visualized with chemiluminescence solution.

### 2.8. Patients and Tissue Specimens

Human gastric tissue samples (8 pairs of GC and adjacent samples, 11 *H. pylori*-positive, and 12 *H. pylori*-negative GC specimens) were collected from GC patients who underwent gastrectomy at the First Affiliated Hospital of Nanchang University. Gastric mucosal tissues of patients who underwent gastroscopic biopsy at the First Affiliated Hospital of Nanchang University (12 *H. pylori*-positive and 12 *H. pylori*-negative patients with gastritis) were collected. The diagnoses of GC and gastritis were confirmed based on histology, and the diagnosis of *H. pylori* infection was based on immunohistochemistry or culture results. All subjects provided informed consent for obtaining the study specimens. The study protocol was approved by the Clinical Research Ethics Committee of the First Affiliated Hospital of Nanchang University. The gastric mucosal tissues of the mice were derived from C57BL/6 mice infected with the *H. pylori* SS1 strain successfully constructed by our research group [12] (12 in the control group and 12 in the *H. pylori* infection group).

### 2.9. Real-Time Quantitative PCR Analysis

TRIzol (Invitrogen) was then used to extract total tissue RNA, after which SYBR® Premix Ex Taq (TaKaRa) was used for qRT–PCR. The primers used for the detection of human specimens: *β*-actin forward primer 5′-TGACGTGGACATCCGCAAAG-3′ and reverse primer 5′-CTGGAAGGTGGACAGCGAGG-3′; GSTO2 forward primer 5′-TGTGTATGGGATACTGGACTGT-3′ and reverse primer 5′-AGGCATTAGGGTTGTTCTGAAAA-3′; HUS1 forward primer 5′-GAATGCCAGGGCTTTGAAAATC-3′ and reverse primer 5′- CACAATGCGGCTACTGCTTG-3′; INTS1 forward primer 5′-GTCAGGCCAATGAATCGAAAAC-3′ and reverse primer 5′-CGACGGAGAAATGGCTCGT-3′; TMEM184A forward primer 5′-CTACACCGTGCCACAGGAG-3′ and reverse primer 5′-CCGCACAGAGTCGAAGTAGA-3′; TMEM190 forward primer 5′-CCAGACGAAAACGTGCGGA-3′ and reverse primer 5′-GCGAGACGGACTTGGACAT-3′.The primers used for the detection of mouse specimens: *β*-actin forward primer 5′-GGCTGTATTCCCCTCCATCG-3′ and reverse primer 5′-TCGTCGGTCCTTAGACAGTGA-3′; GSTO2 forward primer 5′-AAAGCTGTTTCCGTATGACCC-3′ and reverse primer 5′-CGCTATCAGACATTCCTTGCTTA-3′; HUS1 forward primer 5′-AGCTGAACTTCATCCTTTGCG-3′ and reverse primer 5′-ACGGTAAGACAGGGAAAGTGTT-3′; INTS1 forward primer 5′-GTGAAGGCGCTTCCTCTAGG-3′ and reverse primer 5′-ACCCCAGAGCAATAAAGTCCC-3′; TMEM184A forward primer 5′-AGGCGTGTTTGTATGGACTGC-3′ and reverse primer 5′-CGGGGCGGTATAGGAACGTA-3′; TMEM190 forward primer 5′-CCTGTGGCAGCCTACTCTTC-3′ and reverse primer 5′-TCGTCGGTCCTTAGACAGTGA-3′.

### 2.10. Statistical Analysis

The Student's *t*-test was used for comparison between two groups. *P* < 0.05 was considered statistically significant.

## 3. Results

### 3.1. Identification of DMGs and Enrichment Analysis of the DMGs

**Figure 2:**
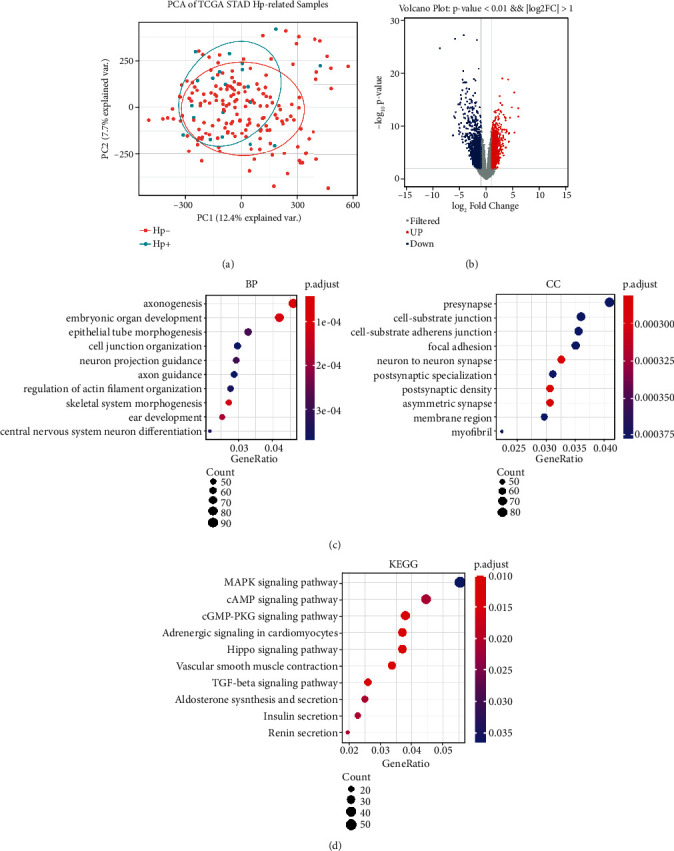
Identification of DMGs and enrichment analysis of the DMGs. (a) PCA of *H. pylori*-positive GC and *H. pylori*-negative GC samples. (b) Volcano map of methylation results in 188 GC samples. Red represents hypermethylation, and blue represents hypomethylation. (c) Top 10 genes from the GO analysis of 2454 DMGs. BP: biological process; CC: cellular component. (d) Top 10 genes from the KEGG analysis. KEGG: Kyoto Encyclopedia of Genes and Genomes.

The methylation data were normalized and standardized. As shown in Figure S1, the normalized methylation data were essentially at the same level, and the normalized data were analyzed for differential methylation. Principal component analysis (PCA) was performed on the selected samples based on the normalized *M* value. As shown in Figure 2(a), the *H. pylori*-positive and -negative samples were not well distinguished. This might be due to the small number of *H. pylori*-positive samples.

According to the screening threshold, a total of 5609 methylation results with significant differences were obtained. The results without corresponding genes and the results of one methylation corresponding to multiple genes were excluded. Finally, a one-to-one methylation-gene relationship was obtained, and a total of 3298 results were obtained for subsequent analysis. The repeated genes were removed, and 2454 DMGs remained (Figure 2(b)), of which 1679 were hypomethylated and 775 were hypermethylated. All 2454 DMGs were displayed in Supplementary Table 1.

The R package Cluster profiler was used for enrichment analysis to investigate the functions of the DMGs. As shown in Figure 2(c), these genes were enriched in the biological process (BP) categories (axonogenesis, embryonic organ development, etc.). The top significant cellular compartment (CC) categories were presynapse, cell-substrate junction, and cell-substrate adherens junction. Kyoto Encyclopedia of Genes and Genomes (KEGG) pathway analysis revealed that the main enrichments were in the MAPK signaling pathway, the Hippo signaling pathway, and the TGF-beta signaling pathway.

### 3.2. Identification of DEGs and Enrichment Analysis of the DEGs

**Figure 3:**
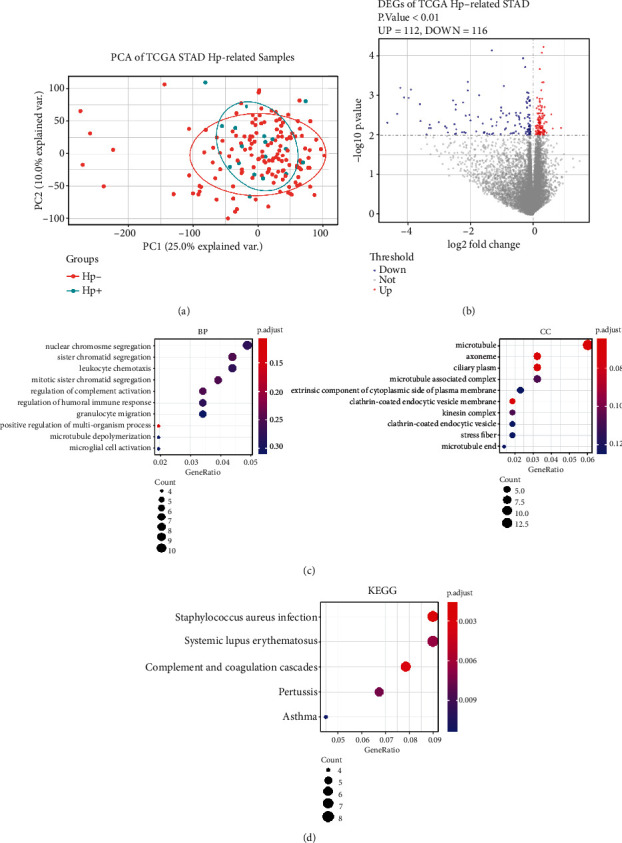
Identification and enrichment analysis of DEGs. (a) PCA of *H. pylori*-positive GC and *H. pylori*-negative GC samples. (b) Volcano map of the difference analysis. The differential gene screening threshold is *P* < 0.01. Red indicates the upregulated DGEs (*n* = 112), and blue represents the downregulated DEGs (*n* = 116). (c) GO analysis of 228 DEGs. BP: biological process; CC: cellular component. (d) KEGG analysis. KEGG: Kyoto Encyclopedia of Genes and Genomes.

PCA results of gene expression were consistent with the methylated PCA results, and the *H. pylori*-positive and *H. pylori*-negative samples were not clearly divided into two clusters (Figure 3(a)). As described in the Methods section, a differential analysis was performed on all mRNA expression levels in the selected samples. According to the screening threshold, a volcano map of the difference analysis results is shown in Figure 3(b), and a total of 228 DEGs (112 upregulated genes and 116 downregulated genes) were obtained. The entire list of 228 DEGs is presented in Supplementary Table 2. Next, we conducted an enrichment analysis on the 228 DEGs, and GO analysis did not obtain statistically significant results, though these genes were mainly enriched in chromatin separation in the BP category and microtubules in the CC category. KEGG pathway enrichment results were mainly related to the immune system, the immune response, and complement pathways (Figure 3(c)).

### 3.3. Screening for Genes with Reduced Expression Caused by Hypermethylation

**Figure 4:**
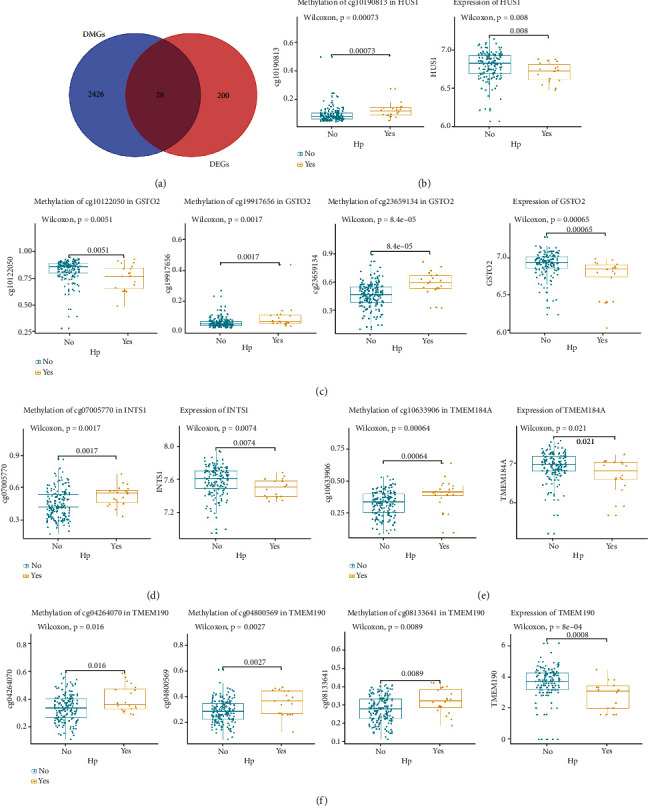
Screening for genes with reduced expression caused by hypermethylation. (a) Venn diagram of the intersection of 2454 DMGs and 228 DEGs. (b)–(f) Genes with reduced expression may be caused by hypermethylation. Box plots of methylation levels and gene expression levels of the five genes (GSTO2, HUS1, INTS1, TMEM184A, and TMEM190) in the two groups are shown. Blue represents the *H. pylori*-positive group, and yellow represents the *H. pylori*-negative group.

To obtain genes affected by methylation, we took the intersection of 2454 DMGs and 228 DEGs, as shown in Figure 4(a). A total of 28 genes were identified, and the functions of these 28 genes are shown in Supplementary Table 3. Furthermore, the methylation sites and the expression of the 28 genes in different groups (*H. pylori*-positive and *H. pylori*-negative) were visualized. Finally, five genes with reduced expression that may be caused by hypermethylation were obtained, namely, GSTO2 (cg10122050, cg19917656, and cg23659134), HUS1 (cg10190813), INTS1 (cg07005770), TMEM184A (cg10633906), and TMEM190 (cg08133641, cg04264070, and cg04800569) (Figures 4(b)–4(f)).

### 3.4. Experimental Verification of the Five Genes

**Figure 5:**
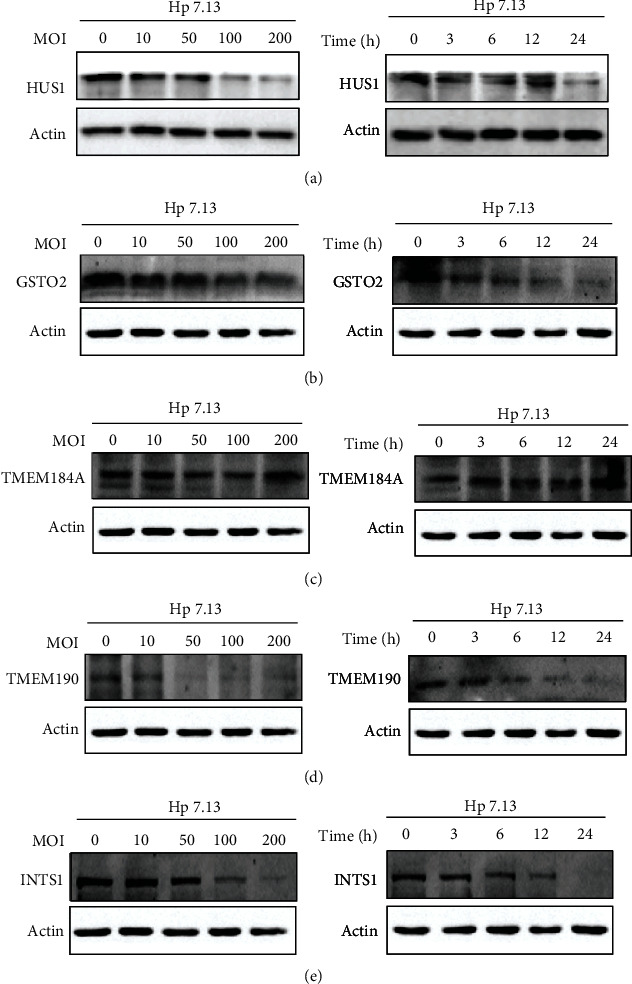
*In vitro* experimental verification of the five genes. (a)–(e) Western blots of the five genes (GSTO2, HUS1, INTS1, TMEM184A, and TMEM190) in AGS cells with *H. pylori* 7.13 infection at different MOIs or at different time points; ^∗^*P* < .05, ^∗∗^*P* < .01, ^∗∗∗^*P* < .001.

The above five genes were selected by comparing the *H. pylori*-positive GC and *H. pylori*-negative GC samples. To confirm the above findings, we cocultured the *H. pylori* 7.13 strain with AGS cells and found that *H. pylori* infection significantly downregulated the expression levels of HUS1, GSTO2, TMEM190, and INTS1 but not TMEM184A (Figures 5(a)–5(e)). This is consistent with the previous analysis results.

**Figure 6:**
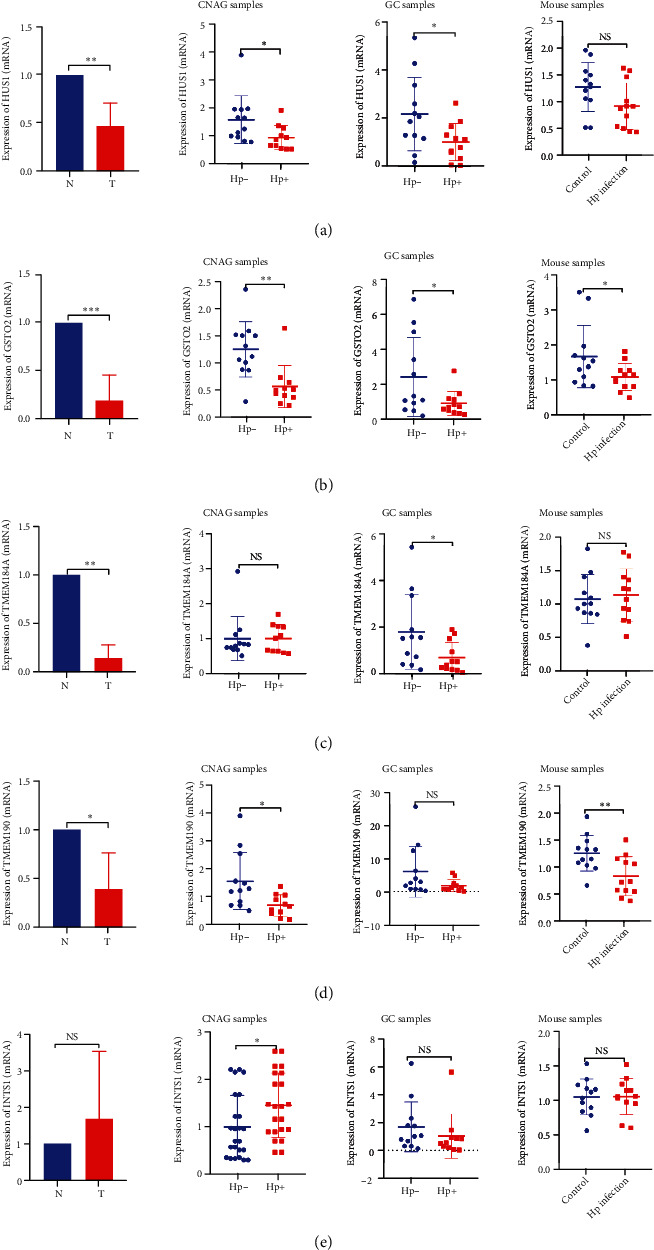
*In vivo* experimental verification of the five genes. (a)–(e) Expression of the five genes in GC and adjacent samples (*n* = 8), *H. pylori*-positive (*n* = 11) and *H. pylori*-negative (*n* = 12) CNAG samples, *H. pylori*-positive (*n* = 12) and *H. pylori*-negative (*n* = 12) GC samples, and *H. pylori*-infected (*n* = 12) and uninfected (*n* = 12) mouse samples. Hp-: *H. pylori*-negative; Hp+: *H. pylori*-positive. ^∗^*P* < .05, ^∗∗^*P* < .01, ^∗∗∗^*P* < .001.

We also examined the expression levels of these five genes in eight pairs of GC and adjacent samples and found that the HUS1, GSTO2, TMEM184A, and TMEM190 mRNA levels in GC samples were significantly lower than those in adjacent samples (*P* < 0.05). We collected *H. pylori*-positive and *H. pylori*-negative chronic nonatrophic gastritis (CNAG) and GC specimens and found that HUS1 and GSTO2 were significantly downregulated (*P* < 0.05) in the *H. pylori*-positive group of CNAG samples and GC samples. The mRNA level of TMEM190 was also downregulated in the *H. pylori*-positive group (*P* < 0.05), even though the difference was not statistically significant in the GC samples. In addition, we verified these genes at the animal level and found that the mRNA levels of GSTO2 and TMEM190 were significantly downregulated in the gastric mucosa of *H. pylori*-infected mice (*P* < 0.05) (Figures 6(a)–6(e)).

## 4. Discussion

Abnormal DNA methylation in the gene promoter region is believed to play a crucial role in GC tumorigenesis [13, 14]. The pathogen *H. pylori* is known to be closely related to GC. *H. pylori* may promote carcinogenesis by inducing abnormal methylation of gastric epithelial cells [15]. However, it is necessary to conduct further research to clarify the detailed molecular mechanism of the abnormal promoter methylation caused by infection with this pathogen.

This study comprehensively analyzed the DNA methylation and RNA-seq data of *H. pylori*-positive and -negative GC samples to investigate the changes in DNA methylation patterns present in *H. pylori*-associated GC. A total of 2454 DMGs and 228 DEGs were identified in this study. To study the roles of epigenetic DNA changes in pathways, the DMGs and DEGs were comprehensively analyzed. The enrichment results of DEG functions and pathways were mainly related to the immune system, the immune response, and the complement pathway. It is known that immunity is involved in the generation and development of GC [16]. The enrichment results of gene functions and pathways corresponding to DMGs were mainly concentrated in various signal transduction pathways, such as the Hippo signaling pathway and the MAPK signaling pathway. The Hippo signaling pathway is considered to regulate cell proliferation, programmed death, and cancer formation [17]. Previous research has shown that epigenetic changes induced by *H. pylori* in GC contain abnormal methylation of components of the MAPK signaling pathway [15]. These results suggest that abnormally methylated genes may lead to the destruction of core cancer signaling pathways and play significant roles in the occurrence and development of *H. pylori*-associated GC.

Five genes that may be downregulated by hypermethylation were identified as GSTO2 (cg10122050, cg19917656, and cg23659134), HUS1 (cg10190813), INTS1 (cg07005770), TMEM184A (cg10633906), and TMEM190 (cg08133641, cg04264070, and cg04800569). Studies have shown that the HUS1 gene is involved in DNA damage repair and apoptosis and has a clear relationship with the occurrence and development of tumors [18]. In our study, it was also found that *H. pylori* infection can significantly downregulate the expression of HUS1. The expression level of HUS1 in GC tissues was significantly lower than that in adjacent tissues. Studies have shown that *H. pylori* infection decreases the expression of GST. The mechanism may be due to the deletion of the GST gene caused by *H. pylori* infection or one of the pathogenic factors inhibiting the activation of the active site of GST, thereby weakening the protective effect of GST on the gastric mucosa [19, 20]. The genetic polymorphism of GSTO2 is closely related to the risk of GC [21]. Similarly, this study also found that *H. pylori* infection downregulated the expression of GSTO2, and the expression of GSTO2 in GC tissues was also significantly lower than that in adjacent tissues. This is consistent with current studies. In recent years, it has been reported that INTS1 plays important roles in the DNA damage response, perinuclear dynein recruitment, adipocyte differentiation and maturation, hematopoiesis, primary cilia formation, tumor development, and virus microRNA formation [22], while its role in the pathogenesis of *H. pylori* infection has not yet been reported. In our study, *H. pylori* infection significantly downregulated its expression. However, no significant difference was detected in the expression levels in GC and adjacent tissues, which may be due to the small sample size. As a result, further experiments are needed to explore its role in *H. pylori* carcinogenesis. TMEM184A is a conserved transmembrane protein that is related to the sex determination of germ cells and the interaction between germ cells and somatic cells and participates in the anti-inflammatory response [23]. This study shows that its expression in GC is significantly lower than that in adjacent tissues. However, its relationship with *H. pylori* infection needs to be further verified. TMEM190 is a small transmembrane protein containing a trefoil domain that was previously discovered through proteomic analysis of mouse sperm. However, the biological significance of the molecule is still unclear [24]. This study found, for the first time, that *H. pylori* infection can downregulate the expression of TMEM190, and the expression of this gene in GC was significantly lower than that in adjacent tissues.

In the process of cancer occurrence and development, DNA methylation changes may be detected earlier than other types of prognostic markers, with higher clinical sensitivity and dynamic range. Therefore, the combination of these candidate genes with other genetic and transcriptional events of *H. pylori*-associated GC will help to improve the accuracy of prognosis prediction. However, this study has some limitations. First, more independent datasets are required to further validate the prognostic value of the genes. Second, additional experimental studies are needed to understand their functional effects.

## 5. Conclusion

In conclusion, our study reveals five genes affected by methylation using public expression and epigenetic datasets. We further found that HUS1, GSTO2, and TMEM190 can be downregulated by *H. pylori* infection, and that their expression levels were lower in GC. We demonstrated that HUS1, GSTO2, and TMEM190 may play an important role in the pathogenesis and carcinogenesis of *H. pylori* infection and may be related to the methylation process. This study not only increases the understanding of the potential molecular mechanism of *H. pylori*-associated GC but also proves the role of abnormal DNA methylation in *H. pylori-*associated GC.

## Data Availability

The original data used in this study were all from the TCGA database (https://cancergenome.nih.gov/).

## Supplementary Materials

Supplementary Materials Figure S1: normalization and standardization of methylation data. (a) Unnormalized TCGA GC methylation data. (b) Normalized TCGA GC methylation data. Supplementary Table 1: list of 2454 DMGs. Supplementary Table 2: list of 228 DEGs. Supplementary Table 3: functional roles of the 28 genes.
