# Supplementary material for: Discovery and Validation of Novel Methylation Markers in Helicobacter pylori-Associated Gastric Cancer
Source: Dis Markers. 2021 Dec 8;2021:4391133. doi: 10.1155/2021/4391133 (PMC8674074; doi:10.1155/2021/4391133)
Supplement: Supplementary Materials — Figure S1: normalization and standardization of methylation data. (a) Unnormalized TCGA GC methylation data. (b) Normalized TCGA GC methylation data. Supplementary Table 1: list of 2454 DMGs. Supplementary Table 2: list of 228 DEGs. Supplementary Table 3: functional roles of the 28 genes. [file 4391133.f1.pdf]

Figure legends:

Figure S1. Normalization and standardization of methylation data. (A) Unnormalized TCGA GC methylation data. (B) Normalized TCGA GC methylation data.

Supplementary Table 1. List of 2454 DMGs.

Supplementary Table 2. List of 228 DEGs.

Supplementary Table 3. Functional roles of the 28 genes.

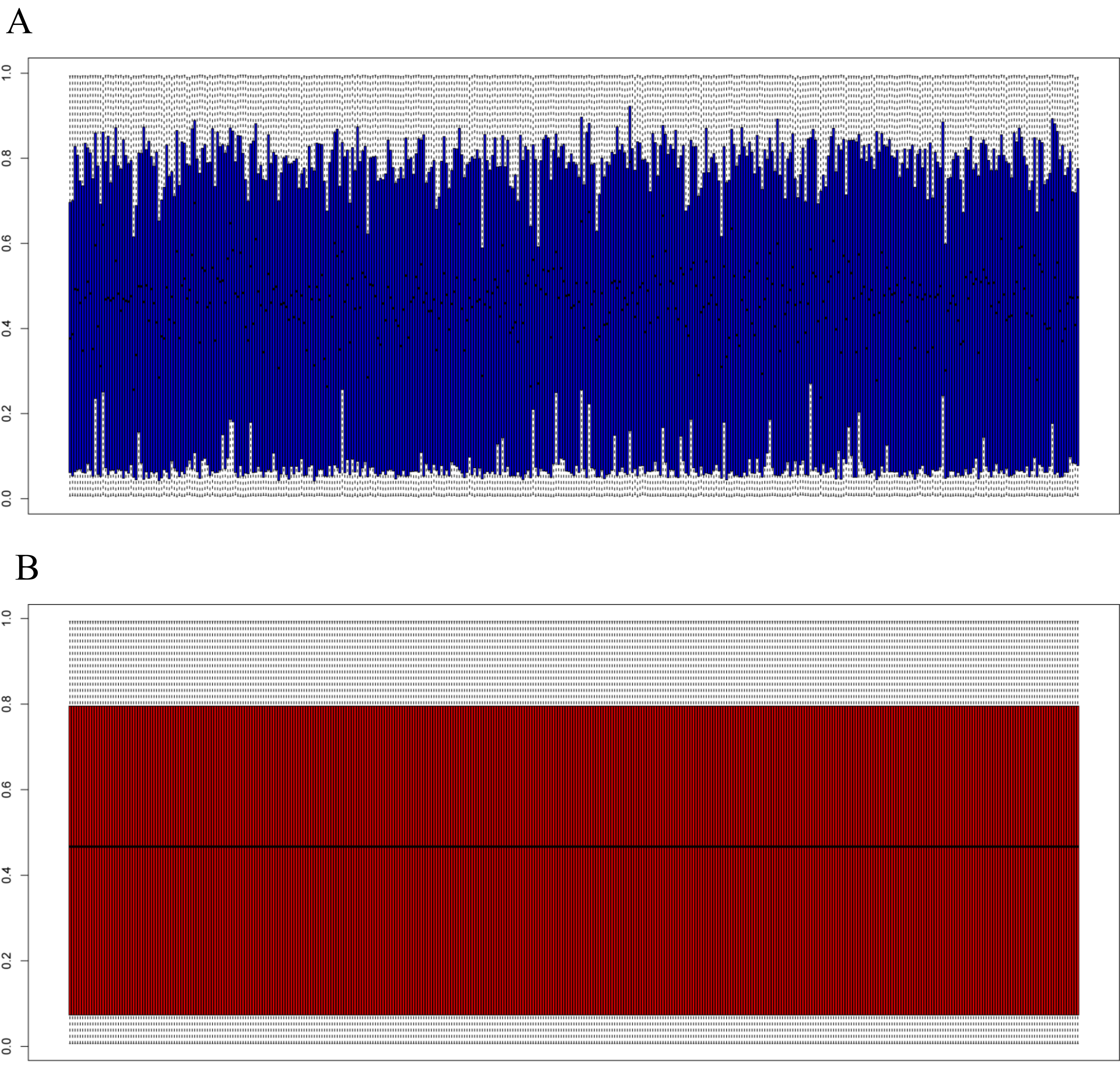

Figure S1 Normalization and standardization of methylation data

Genes
ZXDC
PSMD3
NOMO2
RNF144B
EHBP1L1
PLEC
DIAPH3
RERE
UVSSA
SYPL1
TNRC6A
CNPY3
TP53BP2
UBE2V2
ASXL2
ADPRHL2
C1QBP
RNF19A
TCP11L1
ZNF316
RPRD1A
THA1P
C18orf8
AKAP8L
TCFL5
LEMD3
ALKBH8
IWS1
ZNF324B
ABT1
FAM20B
KLF10
RP11-1094H24.3
NTPCR
HDDC2
COQ5
ASB13
POLM
MRPS31
PRKCD
RP4-550H1.7
ACSL3

CCDC71
SCFD1
COLGALT1
ACLY
HP1BP3
MDN1
LSM5
DHX16
FKBP8
C1orf198
TPI1
HSPH1
ALS2
ARPC2
DHDH
KATNBL1
TAF10
ZNF318
STRIP1
YWHAG
ZNF384
RALGAPB
TWISTNB
GOLGB1
CDAN1
TWF1
MLX
TARBP1
ATN1
TGIF1
OSGIN2
CYB5R4
LANCL2
SLC43A2
CHAMP1
GABBR1
RRP7B
KDELC2
ADD1
MYBL1
PDZD8
PRSS16
YWHAZ

SECISBP2L
ASPHD2
RAB1A
MAP3K8
ATP5G3
RPH3AL
MTFMT
SLMO2
RPS27
PIP5K1A
CCDC138
CDC27
USP49
PIGU
MTR
LPCAT4
UBE2K
PNKP
SIKE1
AAR2
SSC4D
IL1RAP
SLMAP
PRDM15
BRAT1
SMYD5
PIP4K2B
INPP4A
SKIL
RUFY2
ZNF621
CDV3
SPATA20
SLC9A8
RNF168
HIP1R
GANAB
SPATA2
BCL3
SPTBN1
VPS37D
ZNF623
C17orf70

RANBP9
MKNK2
ADCK5
HNRNPL
TNIP1
HELZ
ZFPM1
AKR1A1
FAM195B
GDPD1
TTC27
UNG
ATP6V0A2
PIP4K2C
PCGF2
TRA2A
KBTBD2
ARID3A
SENP2
FAM208B
KB-1460A1.1
KLHL25
SAC3D1
KIAA2018
GLRX3
RBM17
DEGS1
SLMO1
CDK2AP1
ITSN2
NR1D1
ATP1B3
UBE2D1
DDR1
SMC3
SNX8
HSPA9
SELM
PCNP
PHTF1
RAB23
RPS13
E2F4

PICK1
BCL7B
PFKL
NR2C2AP
USF1
B3GNTL1
VGLL4
EID2
ATF3
MEGF8
MSTO2P
ITPKB
CENPU
ADSS
DNAJC21
NDUFS7
COPRS
CCDC134
PRMT9
PPOX
ATG7
CALR
FAM222B
NPTN
PPP2R1B
DIS3L2
FRA10AC1
TTLL1
TCEA1
SHMT1
PHF3
NOP2
SF3B5
STX7
CDADC1
OTUD3
RP11-69J7.1
NF2
SLC25A23
CDR2L
MUM1
PDE12
EEA1

NR4A1
DLST
VPS54
DDX41
DCP2
IFFO2
PLEKHH3
SSBP4
C19orf25
MMD
MLK4
STK35
APTX
BCL2
FH
TRABD2A
NOL11
TLE3
CCNI
KNSTRN
AFG3L2
FASTKD1
CARM1
KIAA0907
TMEM14C
PTPN12
NUPL2
VASP
ARL2BP
GNA13
PEPD
PLEKHM1P
LENG9
TICAM1
ATG5
DESI2
CNNM3
WEE1
MAPK9
DYNC1LI1
SCAMP2
PDIK1L
RALGAPA2

BRD3
NAA30
P4HB
SPECC1
GNAI2
MAD2L1BP
VPS72
DHX8
RCOR3
LHPP
UBXN11
CD55
UBXN1
ZSWIM5
SPRED2
NR4A2
PAIP2
RB1CC1
HLA-E
UHRF1
ARL14EP
CXADR
RP11-756K15.2
TSPAN13
ZNF770
SUPT5H
EIF4H
TRAF3
VPS25
CMTM6
ZNF354A
SLC16A13
TAMM41
DCUN1D4
RABIF
PLEKHB2
IRF2BP1
STK17A
PYGB
LMO4
RP4-536B24.4
TBL1XR1
ANP32B

TSG101
OPN3
SPAG9
TC2N
RREB1
PLEKHA3
GTPBP3
SPTLC2
FZR1
CCDC7
CDC42SE2
SLC25A36
CPPED1
ERO1L
R3HDM4
RPRD2
ATP8B3
CRLF3
IARS2
PNPT1
ARF1
GALNT2
KANK2
PPP6R2
FBXO9
CDK8
FBXW11
DHRS3
SMAD6
C1orf53
TSNAXIP1
TAGLN2
ACSS1
RNPEP
TGFBR2
ZNF235
MCM3
NDUFC1
ABI2
TMEM160
KMT2A
ST6GALNAC2
VTA1

KDM1A
LINC00620
XYLB
SMG9
CHRAC1
ASPSCR1
PDCL3
CUTA
IKZF4
POLRMT
AC004967.7
MRPL33
MSH6
ARID5B
SLC39A10
STIM2
TTLL4
PUM2
PPP4R4
JAGN1
PSMA7
RPE
CDC16
WBP2
ABCB10
DDX55
RRM2
PSRC1
R3HCC1L
ZFP36L1
SLC26A2
HERPUD1
MAP2K3
LSG1
SNX3
RARA
RPS7
DUSP2
TMEM161A
ILKAP
CSNK1D
EPS8
CCNL1

EDN1
RP11-88E10.4
RAP1A
DUSP7
ZNF683
LRRC41
TMEM70
SSX2IP
TTL
DBNL
DDX17
JADE1
PTBP2
TCTN3
PHLDA2
LCLAT1
RPL29
TMED9
ALMS1
UBTF
CNN2
C16orf72
NADK
VPS26A
ATP2C1
PDCD2
EPRS
CREBL2
MTERF4
CHMP6
SLC41A1
FAM98C
CALM1
ARHGEF11
MOGS
AC004895.4
DHRS13
NECAP2
ANGEL1
TRIM27
FAM208A
HNRNPC
NUS1

SLC35F5
PRC1
ABHD17A
COPS4
MRPL42
PSMA1
AP1G1
PIK3CB
ANKRD40
TTC1
MAST3
EIF1AX
ZNF557
KIAA0141
CLSPN
PTK2B
GRN
HAX1
RGL2
CD47
TOMM34
DTWD2
ZBED4
COPS7A
USP45
DPP8
SENP6
SLC35E3
TACO1
DHX30
HDAC7
AP1M1
POLR3B
DDHD2
BUB1
UHRF2
FOXN2
LBR
MDM4
S100A6
CRADD
FARSB
TRMT10C

PFKP
RABEP2
CLPTM1L
ASNA1
GADD45B
ADAR
ESCO2
SUPT20H
RAPGEF2
SERPINB1
GRHL1
RNY3
EXOC7
C6orf226
KIAA0355
GTF3A
CNP
DDX60L
BRIP1
HJURP
CENPC
CCDC61
ATP2B4
ZNF440
SAMD1
TMEM87B
PLCXD2
KIDINS220
FKBPL
CALU
DTNBP1
TMOD3
RPS6KB2
INPP5F
TET2
CTD-2311M21.4
VPS36
RP11-91I20.2
LONRF1
PSMD4
G6PC3
USP8
MCL1

SUN1
LIPG
TM9SF3
HPS3
CNOT1
C19orf24
QRICH1
ZNF224
PHF14
E2F6
INTS8
CHSY1
ACTN4
EIF5A
CACTIN
SRPK1
STEAP3
POLD2
SLC27A1
RCBTB1
ATG14
SPOP
CNIH4
ESPL1
SEPW1
TRIM26
MEF2A
NDUFA10
CANX
AK7
RAB28
JADE2
SRD5A3
XXYLT1
XXbac-BPG170G13.32
PPP4C
DPH2
SDR39U1
GTF2B
ZNF766
RICTOR
RCAN3

EIF4EBP2
PAPD4
ZNF852
MTFR1
PREP
MTO1
HIBADH
COQ10B
BIN1
HMGXB4
UBTD2
ATL3
GLO1
MALSU1
LACE1
BCLAF1
XXyac-YX65C7_A.2
USP22
EIF3H
ZNF644
KDM5B
RAP1B
MTRF1L
AC005592.2
FANCE
DCP1A
C17orf62
KIAA0232
PDXK
KCNH8
PSEN2
RNF26
HSPBP1
MED13
MAPK1
TJP2
TRIM68
ARSA
NDUFS5
PRDX3
CTBS
LEMD2
MLLT1

MED30
NDUFB7
TBCE
BAG3
MAN2A2
MOV10
HIST4H4
MFSD5
VAMP4
TOR2A
SIAE
SMAD1
INTS10
CUX1
HMGN2
CCNYL1
MED27
CUEDC2
SGMS1
CHD8
WIZ
TMEM191A
ZNF678
RTTN
RP5-1119A7.14
CDH24
PJA2
KDELR1
CYP20A1
IRAK1BP1
SLC31A2
UBA3
AHDC1
TEAD3
CREBRF
BTRC
ITGA6
GPR156
ERAP1
HES1
RBBP5
ADSL
SPC25

COL1A1
NEIL2
GMCL1
USP1
CST9
PREB
PCTP
ZNF490
DHRS11
MED1
CD6
ERI1
GTF2I
KIFC1
EMC2
KCTD3
TRPM7
SRP72
PPP2R1A
PTGES3
SMAD3
TRUB1
HNRNPA2B1
C19orf12
CDC73
ATRN
LSR
FBXO46
CTTNBP2NL
DCTN4
CITED2
TMEM230
ZHX3
KDM3B
TPX2
MAPKAPK3
LYPLA1
GBF1
STAT3
RBM19
LINC01183
STK38L
C17orf85

IGSF8
RHEBL1
SIRT4
ZMIZ2
CENPA
ETV3
RP11-318A15.2
ZUFSP
CYB561
RBM6
NEK7
DHX34
AP2A1
CHD9
PSMA5
HCG14
OXSR1
SRM
ANGPTL6
TRAF3IP2
GATAD2A
MRPS15
POGK
FNBP4
ARF6
DBF4B
CNOT10
BIRC2
SEH1L
MTHFD1L
GHR
ZNF784
ATP1B1
KLK10
SLC2A4
SNX1
MAP4
FBXO7
ZNF775
HBEGF
CEP162
ABL2
TIPARP

PLAUR
RPL24
SNRPD1
LRFN3
FADD
CFAP36
B4GALT2
RAB12
ZNF148
SRBD1
GORASP2
ZMYND11
DNAJB12
SDHAF1
LINC00282
NFE2L3
ZFYVE16
ITPK1
ETF1
SLC38A9
TAOK3
ADAMTSL4-AS1
PPP1R12C
RNF216P1
BCAS3
OLFML3
NUDT9
TOP2B
MRPL55
DPF2
RPL27
DNAJC13
MAGI3
SKAP2
UBXN2A
RBM4B
SHROOM3
ITPRIP
GDF5
BUB1B
PDLIM5
TNFAIP8
DAXX

KLHL24
CBX8
BPGM
COMMD9
ZNF837
SMUG1
PHF1
ZNF519
IMPDH1
CHCHD1
ZBTB43
KLHDC2
RPL13A
MORF4L1
SLC39A14
TADA1
AHCYL1
ADRB2
C10orf76
ST14
CTD-2377D24.6
UBB
POC1A
BIRC6
FXYD3
PPIA
GMFG
NFKB2
RASL11A
METTL20
PTPRG
AHCYL2
MICAL3
PBX1
DUSP5
OCEL1
THAP1
IGSF9B
MET
MEX3B
ZSCAN10
AHSA2
MARK4

CYP26A1
FRAT2
ZNF202
TIAL1
RP11-156P1.3
ADCK4
DEF6
GIP
CACNA2D2
FCER2
MYOM3
HNRNPUL1
EXPH5
ZNF805
FER
IPO8
CIC
PTPN21
WDR47
AC053503.4
MARK2
DNAJC15
EFCAB12
EBAG9
RP11-278H7.4
POU2F2
SLC25A37
CD83
BCL6
HUS1
MBIP
MIDN
CACNA2D3
ATAD5
QSOX1
TCEAL1
STK40
DGCR14
RP11-190A12.9
PPP2R5A
SRL
PERP
C4orf3

ITGAE
UBE3A
ZNF614
ZNF507
NR5A2
MIER2
GCC2
CCNY
MN1
SMURF2
CMC1
PODXL
C2orf73
NPAS1
MME
RTN1
DRG2
MUCL1
SHOX2
SEC61A1
FSTL4
HEATR1
ADAM22
ACP6
SNRNP70
CBLC
ECE1
COL12A1
ATR
SLC17A7
SALL3
FNBP1L
ZFYVE27
SYDE1
USP18
CBFB
POPDC3
CSRNP3
RANBP17
WNT9B
STAC2
ZNF256
CSMD3

LOX
CLYBL
LRIG3
ATIC
TMC8
PDGFRL
NBR1
DOK7
RP11-209K10.2
TM6SF1
NEUROD2
RPL39L
MOCS1
EVL
AKR1B10
HMSD
TRIM56
GALNT7
CLN5
IMPA2
WDR59
EHMT2
GNAI1
CNIH3
APBB2
SYT14
RP11-95M5.1
CARS
RMDN3
PACSIN2
BPIFB1
ATP6V1E2
NOSTRIN
EBF3
FBXO17
NCF4
CDH23
RP11-433J22.3
TPD52
ANO4
GFPT2
SLC17A6
ITGB2

MYO9B
RPS15
GAD2
GOLM1
AKAP7
PKN1
ANO5
RBM24
LCK
CUX2
C10orf67
FBXL2
RGS4
CDC42EP1
KIF19
SSBP3
NDFIP1
VTRNA1-2
FKBP14
NRP1
DPH6-AS1
PLA2G3
RN7SL208P
RCC1
APC2
ZC3HAV1L
SMEK1
FHIT
CTD-2319I12.3
KCNK2
C1orf127
RP11-180P8.1
MIR4458HG
PXK
LINC01158
B4GALNT4
ADCY4
SORBS2
LPAR5
RP11-467N20.7
SLC5A1
NKIRAS2
EMILIN3

ACTL8
TGOLN2
SRA1
SULT2B1
AGTR1
NEUROG1
ZNF311
CSMD1
LINC01299
RBM47
CHST7
TULP3
CNRIP1
MAF
ADCY3
CABLES1
MMEL1
AC084262.2
KATNAL2
MIRLET7BHG
ZNF354C
RP11-169E6.4
RP5-912I13.1
FOXE3
F2
HDAC5
PALMD
AOC4P
CNPY1
EGR4
C6orf99
UROD
SLITRK3
NWD1
PCLO
PDIA5
ESRP2
BRMS1L
HSD17B13
RYR1
POU2F3
NDST4
ATP5G2

LRRC7
ERN1
INSM2
EMX1
TECPR2
LUC7L3
ADCYAP1R1
CLDN4
TMEM51
TTC33
GRIK5
PTBP1
PAK7
CDPF1
SNX31
SKOR1
GPATCH4
SCARA5
SH3TC2
ANXA2
RASGRP2
FOXP1
OSBPL10
INPP4B
NKX3-1
STMN4
FILIP1
FIGN
NCKAP1
CDK15
CTSH
DFNB31
COMP
TMTC1
IZUMO2
PPP5D1
RP11-65F13.3
LINC00910
HRC
XYLT2
CETP
ME3
GARS

DIP2C
DIRC3
RNA5SP40
PLEKHG4B
GP2
DOC2B
RMND5B
CBX7
ALG14
SCTR
ZNF536
CALD1
MUC5B
FRMD4A
RP11-230G5.2
UPF1
RP11-462L8.1
PRR16
GFRA1
CCDC12
SLC22A2
LASP1
VPS13D
HS3ST3B1
RASL11B
RP11-346L1.2
MANSC4
IFI35
BICC1
NANOS2
RPS26P49
CASZ1
MBTPS1
SLC9A2
CWH43
HTR1E
OLFM3
44447
TENM4
SLC25A21
EDAR
MCU
LINC00483

HS3ST4
LRRCC1
KCNJ6
PDE6B
RP11-734K2.4
GRIK2
COL14A1
PAX7
MT2A
PRKG1-AS1
CSMD2
KCNJ8
MEX3D
LHX2
LILRA1
CACNA1F
KIF6
FRZB
MAP4K4
NKX2-8
TLL1
STAB1
PRKG1
RP11-174G6.1
CDA
STC2
KIF17
MORN1
RDH8
PDE1C
NMRK2
SH3BP4
HAMP
PAX2
AOC3
RP11-170M17.1
KCNJ11
RIPK1
METTL11B
PLXNB1
CPAMD8
MDFIC
CCDC126

PRAMEF11
AC011286.1
KIAA1683
NIT2
ARHGAP36
NUPR1
SDC4
NAALAD2
ISLR2
WNT7A
EFCAB2
PADI3
COASY
PPM1L
GALNTL6
LHX4
WSCD1
CEP85L
CTC-537E7.2
RIOK3
FAIM3
SNTG1
SMCR8
SLC27A4
EOMES
LDHD
FOXI1
CCDC33
LIN28A
ARHGAP12
MAGI2
NTM
LINC00634
ANKRD34C-AS1
HS3ST2
FOXA2
FOXG1-AS1
AP001347.6
CD40
RP11-520P18.5
PROB1
ZNF324
PPP2CB

COPZ2
HBM
LSP1
LRRC71
MYL7
GCFC2
TMEM184A
VNN3
FAM19A2
GULOP
AGBL4
RELB
CCL28
EZR
MYT1
EBF1
ADCY6
RP11-158J3.2
ZNF804B
FGF8
KLK11
GUCY1A2
AKR7L
RP11-1220K2.2
PLCH1
PLA2G2E
RGR
KCNK12
CSF1R
SLC27A5
SLC16A7
ADRA1B
GPR149
IGFBP6
CKAP2L
UCN3
GCNT3
MATK
RNF128
RP11-13J10.1
MSLN
FAM46A
NFASC

SRPK3
HIVEP3
MAST4
TPO
ZSWIM4
AJ003147.9
ITGB7
PCYT2
C2orf40
ERICH1-AS1
ANK1
ZNF350
TMEM190
TMIE
FADS6
WDFY3
RALA
C1orf229
LINC00511
AUTS2
ZNF880
TRIM29
CTD-2207P18.2
KBTBD11-OT1
RPS5
IRX4
DOK6
DNAI1
FFAR2
SYN3
PSMG3
RP11-19E11.1
DNER
FAM71E1
LHX5
TTF2
PRDM16
PLOD2
KLC3
SLC38A10
EPHA10
CPNE4
RP11-413P11.1

RSPO3
RP11-324C10.1
RIN3
ATAD3C
TRIM67
ANGPT4
SYBU
SLC6A1
OCIAD2
NLK
RP11-202A13.1
FAM167A
MUC13
CEACAM20
LGALS4
PCSK4
RP11-410D17.2
ADCY7
CLDN5
PRDM14
BNIP3
CDH8
RP11-624M8.1
RP11-148L24.1
AKAP12
EXOC3L1
C5orf66
GABRA1
ABLIM1
RSPO1
SPACA3
ARHGAP20
SFMBT2
AC073343.13
RP11-353N14.5
CTD-2281E23.2
KRT5
SLC45A3
ANKRD24
KIAA1211L
LINC01342
RP11-1049H7.2
MUC4

PTH1R
ANKDD1B
ANKFN1
ANKRD34B
SYT6
MMP2
SDK1
CCR7
NOVA2
KLK1
BRINP3
FGFBP1
RP11-46C24.3
RP11-1263C18.1
NEGR1
SLC2A1
PLCD3
C1QL2
KCNV1
DPYSL4
IL1R1
CRIM1
COX7A2L
GUCA2B
PSD
SLC13A3
SCRN2
TMEM132E
TJP3
RIPPLY2
ZNF677
OSBPL2
GABRD
KCNJ9
HMX2
PITX2
RP11-305P22.9
PITPNM2
VWA3B
HLCS
BTNL8
KLK4
TNNI1

MYO3A
HCRTR1
GRP
CPLX2
EML1
PHYHIP
ARID1B
ZBTB16
TDRD1
NCL
COL22A1
IFT81
SORBS3
SDR16C5
KCNS3
SLC18A1
ZFP82
LINC01107
TCHH
C1orf86
TRPM3
UBXN4
CTB-35F21.1
TARID
DOCK5
OPCML
MYOD1
HCK
C14orf105
CNTN4
LHX1
VGF
CADPS
CHRDL2
SLN
GIPC1
SLITRK1
B3GALNT2
ZNF503-AS1
GYS1
LINC01163
NOBOX
CLDN11

CHRNA7
P2RX2
SST
ERICH3
PGD
FAM162B
ZNF300P1
TMPRSS13
S100A14
FAM159B
RIMS3
POLD3
VSTM4
GSR
SPON2
CHAT
MATN3
RGS20
ZNF43
RNF43
LINC01411
NRCAM
CNGB1
HHLA2
SPON1
SORCS2
ARPP21
DAP3
CCDC40
NUMBL
GDF10
MYOM2
TBC1D5
SLC6A19
PODNL1
KB-1980E6.3
SP6
MICALCL
PRDM13
RP11-396O20.1
NATD1
SYNDIG1
RABEP1

TRABD2B
IL27
OLFM2
ADAMTS13
KIF22
RP11-32K4.1
AKR1D1
NUDT18
ULBP3
RAB11FIP1
ARHGAP27
PDE6G
L3MBTL1
CALML6
SULT4A1
NLRP1
DNAH9
HLA-DOA
ARHGEF1
LPHN2
DNAI2
DMRTA2
NPPB
RASGEF1A
CTSB
LAD1
CYFIP2
RBPMS2
NCAN
BHLHE23
ZSCAN18
RPH3A
AC005062.2
APEH
CARTPT
IL17REL
TCF24
MEI1
GPR6
SLIT2
PLEKHG4
CELF5
AC022431.2

PRKD1
KLB
SECTM1
NPAS3
VENTX
AC005597.1
SLC32A1
RP11-343J3.2
MAOB
MYL4
SPATA41
BARX2
SLC26A5
LINC00599
MDK
KAZN
XYLT1
ELFN1
NMNAT3
SLC22A31
PHF21B
KRT19
DAB1
ABR
HIF3A
CDH5
AC124861.1
NPR3
GUSB
STK32C
ZNF568
LAMC2
THADA
TFAP2D
FSTL3
CHD5
TMEM52
SRRM4
MOS
C20orf203
ARID5A
FAM172A
INTS1

ZNF471
CTXN3
EPPK1
RP11-561N12.5
HMGCS2
TSLP
CACNA1C
AP000997.2
DLGAP3
NDUFA4L2
KCTD16
CROCC
LAMA2
DLX6-AS1
STK32B
ASPG
GPD1L
GRIN2D
ADAMTS2
LINC01097
RP11-115J16.1
RP11-99J16__A.2
UMAD1
RP4-668J24.2
SLC6A20
LINC00535
RP11-457M11.6
TMEM63A
PNPLA7
KCP
GSC
CYP2E1
FAM101A
HYDIN
ALX4
CTD-2582D11.1
CREB3L1
CTRC
TSC22D2
STAU2
GRID1
RP11-497G19.3
NUPR1L

LRRC73
LHX3
RP11-111I12.1
FAM92B
ATP11AUN
TMEM45B
KDR
ANXA4
SHISA9
ZNF282
IGFBP4
ZIK1
FURIN
BTBD17
NHSL1
CD164L2
PSD3
TTC39A
DPCR1
RP11-81K2.1
MOGAT2
TRPM8
GPR37L1
TBX5
WIF1
C1QL1
GYPC
B4GALT7
NRXN2
TAS1R3
VSX2
SHANK1
MPP7
PNLIPRP2
KIAA1217
ABI1
NXPH1
GBAS
CLSTN1
UNCX
SEC14L3
SLC12A5
CDH18

SAG
LFNG
ADCY5
DUSP4
CBFA2T3
PLXNA2
TCF7L2
CDH16
B3GNT7
IRX2
TBC1D9B
LRRN2
ZNF418
ASIC2
LARP1
OTX2
SPAG6
SHBG
EMX2OS
SYN2
HNRNPU
HCRT
PCDH8
EFHD2
ST6GALNAC5
OTX2-AS1
HCG21
TPTEP1
DPY19L1P1
CDH13
SSPO
HEPACAM
ARHGEF12
SLC7A9
EN1
RNASE1
MEGF6
ZBTB7A
BEST4
TRPS1
ENTPD8
AATK
TBX15

DCC
SUN3
CNR1
OSBPL1A
ACAN
OBSCN
RP11-545A16.3
ATP1A3
RP1-117O3.2
SERINC2
SHF
ANPEP
FAM132A
TLX1NB
BAHCC1
RP11-177H2.1
USP36
SNED1
C9orf3
ESX1
MECOM
SYNC
GALNT15
TMEM105
CARD14
BMP4
PHOX2B
NAV1
ST6GAL2
TP73
ITGA8
SNCB
GAD1
GALR1
CEP112
FGFR1
AC096772.6
AC007796.1
RP11-451B8.1
NUAK1
TMEM196
BAMBI
REEP4

HADHB
DPEP1
ROBO3
CDH2
MIR124-2HG
AEBP2
CCDC102A
SLC35G5
SLC6A2
CHST8
FZD9
WDR86
SVOPL
TMPRSS3
GRHL2
CELF2
ZFP42
ASAP2
CPXM2
GABRG2
ONECUT1
KCNC4
RP11-20G13.1
SFRP1
FAM129A
ONECUT3
KRT6A
CAPN5
PGLYRP4
ADAMTS14
PLA2G2F
INA
SCAMP5
LAMC3
DMTN
ZFP41
LRRTM4
BFSP1
ARHGEF4
PROKR2
ZNF704
VWA5B1
SDE2

RP11-718O11.1
FGF5
LIN9
TLX3
RP11-522B15.3
EYA4
LINC00682
PCK1
PHOX2A
KCNMA1
RP5-1180C18.1
COL23A1
UBE2QL1
RP5-884C9.2
NPAS2
SMG7
ATP8B1
MALL
AMN
PYCARD
CHGA
USH1G
CCDC88C
NANOS3
PROSC
SMIM1
CHST12
BARHL2
AHNAK
VAX1
LTBP4
CTCFL
HK3
CRB2
GPR39
CHST2
HOXD10
DPYS
VAX2
EFNA5
ADARB2
ASCL4
SLC2A14

GGH
RNF220
AC079135.1
GDF7
ARHGAP26
AGPAT3
ZC3H14
AP1S3
LBP
IGF2BP3
AQPEP
LINC01169
PACRG
HHIPL1
NAT8L
RBFOX1
FLT4
BBS2
IQSEC1
C1orf141
IL12A-AS1
CD34
LINC00925
PXT1
HTR2C
PPFIA4
HPX
ARL15
KCNT1
UPP1
TRH
NRXN1
DOK2
C1orf204
SOST
DOK3
SLC24A4
RYR2
PODXL2
RP11-54O7.16
D4S234E
RP11-328K15.1
MSMB

TIAM2
NR2F1-AS1
ANTXR1
BCAT2
ERG
RP11-700P18.1
RP11-964E11.2
CENPL
CLNK
STMN2
FENDRR
RP11-135A1.3
CACNB3
GDF3
CTB-57H20.1
C6
CTD-2666L21.1
RP11-65D24.2
BMPER
RP11-385M4.3
PIP5K1C
ADAMTS12
SCNN1D
CRTAC1
PLXNA4
CAMK4
GLYATL2
ZIC1
PTPRN2
SLC35F4
AC009501.4
GLI3
FGD5P1
ZBED9
PHF21A
UNC5A
ZNF350-AS1
COPS5
BFSP2
IGDCC4
NXPH2
UNC5D
TEKT5

TFAP2A
CATSPERG
MAPT
OR2I1P
SYNDIG1L
KCTD9
TRPC5
NCR1
KIF26B
SLC9A3
ZNF702P
LDLRAD2
OR2G6
UBR4
TUBGCP3
PAX6
CD247
TTC6
RNA5SP174
CMTM2
FHL1
MEST
STK3
DPPA5
ESPNP
CCDC170
SIX3
CTD-2314G24.2
ATP10A
NPHS1
CACNA1G
RP11-10N16.3
CLMN
TBC1D8
ITPR1-AS1
PRRX1
HAPLN3
CTD-2357A8.3
RP4-665J23.1
TAF7L
GALK1
KBTBD11
GHRHR

HCG27
SEC14L1
SPATA21
ACTR5
RP11-317F20.3
GFAP
TMEM175
GRM6
BSND
CASC18
ZNF550
CHADL
FUT2
C1S
FAM71F1
GALNS
LINC01551
ST3GAL3
P4HA3
IGLON5
TTC34
RGL3
UBE2O
PDPN
SLC25A27
ZDHHC21
KIAA1462
TGFB2
CNIH1
ROR1
ST6GAL1
LINC00265
DPF3
KCNAB2
TXNRD2
DMRT3
FARSA
CUEDC1
C15orf27
RP11-299H22.3
C1orf162
ZBTB20
SLC45A4

MURC
AC011516.2
IQCH
ELK1
GFPT1
WNK4
FGF12
RP11-649A16.1
GAPT
FMN2
SLC10A6
FAM189A1
TRIM60
CARD11
ECEL1
ZFP57
RP11-753D20.1
PHACTR2
KRT27
PVT1
RP11-26L20.3
SEMA4B
S1PR1
DPYSL3
SNAI3-AS1
ZIC4
SESN2
MCM5
CTBP1
RSPH6A
HECW1
WNT11
SAMD4A
TAF15
AGR3
CA3
SLC2A2
MIR662
D2HGDH
TARS
NFATC1
OTOP3
ENGASE

LINC00111
TBX20
XKR6
CLU
IKZF2
RPTOR
SEMA6B
FSCN1
NDFIP2
HEPHL1
PIAS1
TDRP
LRRIQ4
ERCC1
ZC3H11B
DLC1
UPB1
LINC01060
PRSS36
KPNA6
PPP1R13L
PTPRG-AS1
SALL1
HSPB8
PPP1R36
GMDS
OTP
NR4A3
CALN1
GRB10
RPL35P4
TWIST1
HMGA2
WDFY4
ARHGAP24
RP11-108O10.2
FAM221A
NXN
KCTD15
MS4A15
NOTCH3
HLA-C
ABHD17C

POP1
ALX1
SLC6A5
GLP1R
EPB42
RTN4
DLGAP2
PRLH
RIC8B
C7orf31
SLC5A9
USP42
ARHGAP22
EDNRA
HDAC4
RAD51B
ERMAP
DTX1
RP11-622C24.2
ZCCHC11
TMC6
PDP1
AMICA1
TG
PRUNE2
CIB2
BICD2
SATB2
NEK10
SMPD3
MGMT
ARHGEF10
MINA
ZBTB17
IL20RA
CLUL1
ATP8B4
MAN1C1
NEK1
TMEM237
C16orf95
DNAJC22
PXDN

CACNA1A
ATP6V0E2-AS1
SYNPO
TSPAN18
ANK2
CACNA1I
LINC00402
UQCRHL
BAK1P2
GREM2
SLCO3A1
ANK3
LTF
ZNF528
DST
MTSS1
RP11-503C24.4
PEAR1
CD40LG
SPEG
FTO
FAM187B
MSANTD2
SNX29
NEURL1B
SFRP2
FNDC3B
NDUFAF6
ANKRD11
LRRC74A
DNAJC18
BRAP
ZNF596
DENND5B
LCT
GP1BA
GFOD1
GP9
SLC5A7
LBH
KCNMB3
NSD1
MEIS1

LGALS7
AK4
FAM216B
TRIML2
PRRT1
SUV420H2
TBC1D16
RP11-1E1.2
C11orf97
SLC25A3P1
APLP2
RD3
RP11-227F19.1
LINC01033
FAM160B1
COX6C
RPL22L1
RASAL1
CAPZB
PANK4
SNRPN
RTKN2
SLC9A1
CYTH3
EPHA4
SP8
MYH14
ECHDC2
GSTO2
TTLL10
LINC01138
LINC01210
MPP5
INPP5J
ALS2CL
PPP2R5B
ZNF343
WWOX
PTGER4
SPRYD7
FAM47E-STBD1
DGKH
TBATA

SLC17A2
GDF6
LINC01250
NECAB1
C14orf37
KDM1B
44450
RPS6KA2
PTPRA
LIN7C
RP11-298I3.1
SLC18B1
AKAP13
ATP2B1
ZFYVE28
SMYD1
CFAP46
P2RX3
SC5D
MYO1C
FAM53A
TNFRSF10B
TREM1
SRGAP3
METAP1D
LMF1
ARHGEF16
SHCBP1L
SLC25A48
SLC1A2
HOXB1
KIAA1644
RGS12
IL5
MPRIP
PEX11A
ABCA2
MACROD1
MIR331
LPCAT1
C5orf47
RBPJ
ISM2

GUCY1B2
MRPL48
FYTTD1
CCDC73
MYO7A
RP11-403I13.5
JRK
KIF21B
KLF12
PRKCE
NEU2
FAM20C
CLK4
AP000255.6
HOPX
MYOZ3
JAKMIP3
FCGRT
LARP4B
C2orf43
FOXP3
KLHDC4
RGS3
RP4-680D5.2
TNIK
AP2A2
PLEKHG1
SMOC2
SYCP2
RP11-973N13.2
NEU1
PCDH8P1
GALNT1
RIMBP2
RAB11FIP4
PIK3R2
HEATR4
PDE4C
TLX2
RP11-545A16.4
BCL2L12
BBX
GPC6

KCNS1
ACSF3
HOXC4
TCF20
RPS14
EP400
C6orf136
UBE3D
CDCA2
STK19
DCAKD
RNA5-8SP2
NBAS
EPHB4
ZSCAN12P1
AGPAT4
ARPC1B
IGFBP2
ASCC2
PPP1R13B
AGAP1
RBX1
GPR137
CXXC5
CHCHD3
SMYD3
ZDHHC14
MYO3B
RNF165
ACTR1B
IST1
SERPINE3
CTU1
H2BFWT
CHFR
SKIV2L
RBM5
PASK
CSTL1
PFKFB3
TMC7
ERICH1
PPP2R2D

FAM3A
ERGIC1
ZCCHC14
IZUMO1
GOT2
CAMKK1
DCTD
TTC7B
PTPN14
GRAMD4
GPX5
SCAPER
MCF2L2
NCOR2
RING1
AC073869.20
ZNRD1-AS1
RP11-241I20.4
UNC93A
LDB3
KCNG2
NPC1L1
CTD-2277K2.1
ANKRD9
BOLA1
TNFRSF1B
JUP
BRMS1
FOXN3
ANKRD33B
GSKIP
DOCK1
TAF4
OGFOD3
DNAJC16
OTUD7B
GPN1
IKZF3
INPP5A
ZNF865
VTI1A
GRAPL
IFT140

PALLD
NADSYN1
ECHS1
SDHA
CCDC92
TUBGCP2
FGFR2
TBCD
AC005077.14
CCAR2
PRKAG2
TPD52L2
EIF3B
ZNF688
CYTH1
BANP
GSE1
MTSS1L
FOXK1
KLRD1
AC009963.6
C3
TMEM201
ZFP92
AGO2
B3GNT3
CLIP2
OIT3
ILVBL
WDR88
HTT
CSNK1G2
MAD1L1
HERC2
PARD3
SRD5A1
IFFO1
PEX5
LRPAP1
VPS53
TAOK2
LMNA
CYFIP1

USP20
WRAP73
ZNF516
SGTA
RELT
TSC2
ARMC5
44448
HDGFRP2
PAOX
MYO15A
DGKQ
GAK
CTDP1
KRBA1
C7orf50
MTHFSD
CARS2
JAG2
GRK7
METTL22
KIAA1549
ARHGEF10L
TFDP1
ZC3H18
NRBP2
PACS2
PCNXL2
LRP1
JARID2
ATP11A
PLXNA1
TRIM35
SCAP
KDM4B
SPSB1
RP11-215H18.5
KLHL29
SCRIB
NDRG1
LMAN2
SRRM2
SPATA5

SH3BP5L
ADCY10P1
KLHL36
ACIN1
TYK2

Supplementary Table 1. List of 2454 DMGs.

Genes
IL36A
TPTE
FABP12
ZFP42
OR9A4
RAG2
ZNF729
SLCO6A1
GABRG1
FGF3
PRB1
OR2H1
PRSS56
C20orf85
PROP1
NTSR2
C1QTNF8
SLC22A8
PRB2
CCDC166
PATE4
TFAP2B
LHX3
OR1Q1
CGA
TRHR
PVALEF
LHX1
IFNK
CEACAM20
OTOP2
CSMD3
STMND1
PRSS37
RNF222
WT1
OOEP
TBR1
MYBPH
LCT
SLC25A52
PPP1R27

SLC28A1
PYY
SLC30A10
NLRP9
TMEM269
CA1
TP53AIP1
SLC22A14
C7orf57
PSORS1C2
TMEM190
VIP
SPAG17
SMIM35
CASC1
GNG4
M1AP
DLGAP1
TLX2
CEACAM4
GALR2
ANGPTL3
HIST1H2BF
TTYH1
SAXO2
RPEL1
SLC16A8
CXCL11
SPATA17
CLEC4E
ZMYND10
TRIM34
CFAP43
C20orf144
IL33
CNN1
TMIGD3
PLA1A
S100B
CA14
ACTG2
POPDC2
GPBAR1

LY86
P2RY13
CALHM6
FCGR1A
SYNPO2
LY96
LYRM9
HCST
KIF17
PRAM1
HSD17B14
C4A
GNGT2
VSIG4
MNDA
GSTO2
KCNE4
HLA-DRB5
RNASE6
MGP
ALOX5AP
GPR34
GLIPR1
CSF3R
LST1
AIF1
C3AR1
LAYN
CELSR3
ECSCR
C1orf162
AMIGO2
CD40
ZNF233
RGS1
MS4A7
MS4A6A
MYL9
JAML
KCNJ8
RENBP
PCED1B
SLA

PLSCR4
HLA-DQA1
GIMAP7
TYROBP
HMMR
TAGLN
PIP4P2
KCNJ14
SELENOM
CLEC2B
SOD3
C1orf54
MAN1C1
RARRES2
TMEM184A
NNMT
GPSM3
VCAM1
FCER1G
HACD4
GMFG
C1QA
GUCY1B1
CDC42EP3
ESPL1
IL18BP
GIMAP4
FERMT2
SPI1
TSC22D3
VAMP5
RHOJ
LY6G5C
C1S
RAMP2
TOP2A
TNFSF12
CENPF
SRGN
FILIP1L
ABCC4
SPAG5
KLHL5

TMEM204
C1R
KIAA0895
SERPING1
CALD1
KIF14
CAMSAP3
KIF18B
EMP3
KIF20A
FHL3
RASSF4
EVA1B
DKK3
ADAP1
TPX2
PRR11
DBF4B
IL1R1
LACC1
IGFBP7
PRC1
HNMT
TIMP1
SHTN1
KLHL22
PNPO
GAN
SMIM29
OSTM1
BPNT1
ARID5A
AP1M2
CCDC77
PATJ
NINJ1
CC2D1A
FAM89B
RRP12
PPP6R1
ERBB3
SUN1
PLEKHA8

CEP89
MSANTD3
HUS1
NCAPD2
INTS1
PANK3
ISCU
GABARAP
NSF
ATXN7L3
DNAJC7
LARP4B
EFTUD2
AP2B1

Supplementary Table 2. List of 228 DEGs.

No.	Gene	Full name	Function
1	TPX2	TPX2 microtubule nucleation factor	Importin-alpha family protein binding
2	TMEM184A	Transmembrane protein 184A	Heparin binding, transporter activity
3	INTS1	Integrator complex subunit 1	RNA Polymerase II Transcription
4	GMFG	Glia maturation factor gamma	Arp2/3 complex binding
5	ARID5A	AT-rich interaction domain 5A	DNA binding, RNA binding
6	HUS1	HUS1 checkpoint clamp component	Protein binding, DNA damage checkpoint
7	CSMD3	CUB and Sushi multiple domains 3	Regulation of dendrite development
8	C1S	Complement C1s	Calcium ion binding, identical protein binding
9	KIF17	Kinesin family member 17	ATP binding, ATPase activity
10	PRC1	Protein regulator of cytokinesis 1	Kinesin binding, microtubule binding
11	CALD1	Caldesmon 1	Actin binding, cadherin binding
12	ZFP42	ZFP42 zinc finger protein	DNA-binding transcription factor activity
13	IL1R1	Interleukin 1 receptor type 1	NAD(P)+ nucleosidase activity
14	KCNJ8	Potassium inwardly rectifying channel subfamily J member 8	ATP binding
15	C1orf162	Chromosome 1 open reading frame 162	Integral component of membrane
16	ESPL1	Extra spindle pole bodies like 1, separase	Catalytic activity
17	LCT	Lactase	βeta-glucosidase activity
18	CEACAM20	CEA cell adhesion molecule 20	Immune system process
19	TLX2	T cell leukemia homeobox 2	DNA-binding transcription activator activity
20	CD40	CD40 molecule	Antigen binding, enzyme binding
21	SUN1	Sad1 and UNC84 domain containing 1	Cytoskeleton-nuclear membrane anchor activity
22	GSTO2	Glutathione S-transferase omega 2	Glutathione dehydrogenase (ascorbate) activity
23	MAN1C1	Mannosidase alpha class 1C member 1	Calcium ion binding, protein binding
24	LHX1	LIM homeobox 1	DNA-binding transcription factor activity
25	LHX3	LIM homeobox 3	Metal ion binding, sequence-specific DNA binding
26	TMEM190	Transmembrane protein 190	Protein binding, protein self-association
27	DBF4B	DBF4 zinc finger B	Nucleic acid binding, protein binding
28	LARP4B	La ribonucleoprotein 4B	RNA binding, protein binding

Supplementary Table 3 Functional roles of the 28 genes
